# Case of Fracture of the Anterior Portion of the Os Calcis

**Published:** 1851-05

**Authors:** W. S. W. Ruschenberger

**Affiliations:** Surgeon, U. S. Navy; Philadelphia


*Case of fracture of the anterior portion of the Os Calcis.* By W. S. W. Ruschenberger, M. D., Surgeon, U. S. Navy.

*November 24th*, 1850. Lieutenant W——, (aged 32 years, athletic, weighing 190 pounds), while in a paroxysm of mental aberration, attempted to escape from the U. S. Naval Hospital, New York, where he had been confined as a patient for several days. Between seven and eight o'clock, P. M., he leaped from the top of a wall, which encloses the grounds, at a point where it is fifteen feet high; and alighted upon his soles on the hard, gravel-paved side-walk of the street. At the time, he was in a dressing gown and slippers.

He was unable to rise upon his feet. Some passers-by conveyed him into a neighboring grog-shop, where I examined him about ten minutes after the fall. It was stated, he was insensible when picked up, and that he had swooned after he was placed in a chair. When first seen by me, he was very pallid and faint; he expressed his opinion, formed from a sense of pain alone, that "all the bones of his feet were crushed." Both ankles and feet were contused. Distinct crepitus was heard on moving the left foot. Below the malleolus externus and a little anterior to the axis of the fibula was a tumor or projection, caused by a fragment of bone, estimated to be about a cubic inch in size, which was seized between the thumb and fingers, and moved easily but not extensively beneath the skin. Moving this fragment caused distinct crepitus and severe pain. The toes and metatarsus could be turned inwards, on a plane with the sole, almost to a right angle, owing to a relaxation, or partial separation of the metatarsal from the tarsal bones. There was nothing abnormal observed in the motions of the astragalus upon the tibia. There was no displacement of the posterior extremity of the os calcis; but the patient stated subsequently, that when his consciousness returned, he found the heel bone drawn up out of place. There was no fracture of the fibula or tibia. Pressure posteriorly to the cuboid showed an unusual yielding space in that region.

The patient was conveyed to his bed in the hospital, complaining considerably of pain. Cold water dressings were applied to the feet and ankles; and a fourth of a grain of meconate of morphia in solution was administered.

The bowels had been moved twice during the day.

25*th*. Ankles and feet considerably swollen, ecchymosed, and very painful. Cold water dressings and meconate of morphia at night.

27*th*. Has less pain; passed a better night; seemed quite rational. Continue treatment.

28*th*. Appears to be perfectly sane. Better; persist.

29*th*. Swelling and pain of ankles less; continue treatment.

30*th*. (Sixth day after accident.) Left ankle and foot carefully examined this morning. The anterior and external portion of the os calcis, which articulates with the os cuboides was fractured and separated; the fragment lying below, and projecting externally beyond the malleolar process of the fibula. Firm steady pressure by the thumbs, directed forward and inward, reduced it, the fragment suddenly slipping into place. A roller was turned firmly around the foot and over a compress placed upon the fractured portion. The entire leg and foot were then encased in felt, previously softened, and the whole dressing retained in place by a roller.

*December 2d.* From the instant the fracture was reduced, he has had no pain in the foot; he seems to be of sound mind.

Although unable to bear either foot in a pendent or vertical position without discomfort or pain, Lieutenant W——, at his own earnest request, was conveyed to his home.

The patient, who is restless and restive in disposition, very soon moved about, and has recently visited Washington City; I have been told he is not lame.

*Remarks.* The history of the above case of rare fracture is derived chiefly from notes by Passed Assistant Surgeon J. B. Gould, U. S. Navy, who was associated with me on duty when it occurred. The accident was probably caused by the sole of the foot coming in violent contact with some inequality, such as a small pebble, opposite to the anterior extremity of the os calcis.

About a week after this fracture, a boy of seventeen years of age, weighing probably less than a hundred pounds, attempted to desert, and leaped the wall at the same point. In his case there was severe contusion and sprains of both ankles, but no fracture. He was able to limp away nearly a half mile.

The influence of the meconate of morphia, as far as it could be determined by administration in the above and one or two other cases, does not differ remarkably from that of the other salts of morphia.

*Philadelphia, April 18th,* 1850.

